# 68-year old man with progressive weakness and ventilator dependent respiratory failure: a case report of sporadic late onset nemaline myopathy

**DOI:** 10.1186/s12890-022-01877-4

**Published:** 2022-03-19

**Authors:** Pradhab Kirupaharan, Daniel Kramer, Alan Gandler, Lawrence Kenyon, Ross Summer

**Affiliations:** 1 Jane and Leonard Korman Respiratory Institute, Thomas Jefferson University, Philadelphia, PA 19107 USA; 2 Harron Lung Center, University of Pennsylvania, Philadelphia, PA USA; 3 Department of Pathology Anatomy and Cell Biology, Thomas Jefferson University, Philadelphia, PA USA

**Keywords:** Neuromuscular disease, Myopathy, Respiratory failure, Sporadic late onset nemaline myopathy

## Abstract

**Background:**

Neuromuscular pathologies must be considered when caring for patients with persistent or progressive respiratory failure. Pertinent disease states may involve skeletal muscles of respiration or associated neurologic structures including motor neurons, peripheral neurons and the neuromuscular junction. Diagnosis may require pulmonary function testing, neurophysiologic studies, imaging, and/or muscle biopsy.

**Case presentation:**

A 68-year-old male was transferred to our intensive care unit (ICU) for management of ventilator dependent respiratory failure. Upon further historical review, he described gradually worsening gait instability and muscle weakness, which was previously attributed to vascular Parkinsonism in the setting of known cerebrovascular disease. Upon arrival to our hospital, he was found to have elevated muscle specific enzymes, prompting evaluation for neuromuscular causes of respiratory failure. He was also found to have elevated HMG-CoA Reductase (HMGCR) antibodies. Ultimately, a right quadriceps muscle biopsy was performed and electron microscopy identified nemaline bodies within skeletal myofibers. Given the clinical course and other histopathologic findings, he was diagnosed with Sporadic late-onset nemaline myopathy (SLONM).

**Conclusion:**

The diagnosis of neuromuscular disease in patients with ventilator dependent respiratory failure is challenging. A detailed history of a patient’s clinical course prior to hospitalization is key and may raise suspicion for underlying neuromuscular pathology. Further evaluation in non-critically ill patients may include pulmonary function, electromyography and confirmatory muscle biopsy. Sporadic late onset nemaline myopathy remains a rare disease entity which rarely presents with respiratory failure and lacks effective treatment.

## Background

The diagnosis of respiratory failure due to neuromuscular disease can be challenging in patients with multiple comorbidities especially in the midst of critical illness. Neuromuscular pathologies must be considered and evaluated in the appropriate clinical context. This article reports a case of sporadic late-onset nemaline myopathy (SLONM) which is a rare, acquired myopathy in a patient who presented with ventilator dependent respiratory failure after a hospitalization for failure to thrive.

## Case presentation

A 68-year-old man was transferred to our intensive care unit (ICU) for management of ventilator dependent respiratory failure. While undergoing rehabilitation following a recent protracted hospital course for “failure to thrive,” he suffered an episode of cardiac arrest presumed to be due to aspiration related hypoxia. He was transferred to our hospital due to difficulty being liberated from mechanical ventilation. Despite improvements in cognition and sensorium following his cardiac arrest, he had persistent motor deficits. Upon historical review, his family reported gait instability and substantial functional decline with complaints of progressive weakness over the preceding years. He was briefly evaluated for this four months prior but was noted to have normal motor function (5/5) in the upper and lower extremities. His symptoms at this time were attributed to vascular Parkinsonism in the context of known cerebrovascular disease, however, further diagnostic workup was not obtained. His physical exam upon arrival to our hospital was notable for absent cough and gag reflexes, diminished gross motor strength of proximal hip flexors (2/5) and extensors (2/5) in the bilateral lower extremities and increased tone throughout all extremities. Otherwise, he had grossly normal strength in the distal lower extremities (4/5) and bilateral upper extremities (4/5). He had normal cranial nerve function with preserved sensation to light touch. His outside hospital workup included modestly elevated creatinine kinase (5100 units/liter) and aldolase (58 units/liter) with no prior levels for comparison. Initial arterial blood gas data following his cardiac arrest showed normal arterial partial pressure of oxygen with minimal oxygen supplementation but significant hypercapnia due to a primary respiratory acidosis. Despite ruling out contributory intrinsic lung and cardiac dysfunction, he required persistently high pressure supported ventilation to maintain adequate acid–base status and minimize visible increased work of breathing. With elevated muscle specific enzymes and a history suggesting progressive isolated motor weakness, he was evaluated for myopathic causes of his persistent respiratory failure. Medications were reviewed and longstanding atorvastatin was discontinued. Additional lab work to assess electrolyte disturbances, thyroid function, paraproteinemia and rheumatologic disorders including myositis were all unrevealing. Computed tomography (CT) of the chest, abdomen and pelvis was unrevealing for occult malignancy and concurrent paraneoplastic serology was also negative. Echocardiography demonstrated normal biventricular size, shape and function without valvular pathology. Pulmonary function testing was not performed due to the fact the patient was hospitalized. Swallowing assessment was not done given endotracheal intubation however he was noted to have normal speech and swallowing function during a speech and language pathologist evaluation three weeks prior. Ultimately, a right quadriceps muscle biopsy was obtained. Subsequent specimens were examined using a Leica DM2500 LED microscope with Leica N Plan 20X/10.40 and N Plan 40X/10.65 objectives and Path4K HDMI 4K C-Mount camera. No downstream processing was utilized. Initial histology demonstrated an increased variation in muscle fiber diameter with many degenerating and regenerating fibers along with a minimal lymphocytic inflammatory infiltrate. Electron microscopy identified nemaline bodies within skeletal myofibers. Additionally, he was found to have elevated HMGCR antibodies (IgG 358 chemiluminescent units) in the setting of known high dose atorvastatin use following an ischemic stroke three years prior. However given the consistent histopathologic findings, he was diagnosed with sporadic late-onset nemaline myopathy (SLONM). After excluding plasma cell dyscrasias and HIV, our patient was started on a five-day course of IVIG with intravenous methylprednisolone (1 mg/kg). Due to interval worsening of renal function and sensorium, he was transitioned to lower dose oral prednisone (40 mg) until planned outpatient follow up by a neuromuscular specialist for further evaluation and consideration for further immune modulating therapies. Though the patient was able to tolerate longer durations of pressure support, he remained ventilator dependent at the time of discharge. He subsequently died one-month after discharge at his long-term advanced care facility.

## Discussion

Sporadic late onset nemaline myopathy (SLONM) is a rarely recognized disorder that was first described in 1966 by A.G. Engel [1]. It typically presents after the age of 40 years with progressive weakness, typically in a limb-girdle distribution. However, distal weakness has been observed both in isolation and in combination with proximal weakness. The serum CK level is typically normal although modest elevations similar to our patient are reported. EMG showing myopathic features with fibrillation potentials may also be seen. Therefore, a diagnosis of SLONM must be considered when these findings are present. A monoclonal gammopathy without features of amyloidosis or multiple myeloma (MGUS) is a common associated finding, although the potential role in the pathogenesis of SLONM is not understood [2]. Interestingly, elevated serum and urine kappa light chain levels (81.5 mg/L and 202 mg/L respectively) were detected in our patient, although subsequent evaluation including serum electrophoresis, immunofixation and bone marrow biopsy with fluorescence in-situ hybridization, served to rule out associated plasma cell disorders including MGUS. Additionally, it is important to note that anti-HMG CoA reductase antibodies were detected in our patient who was on longstanding high dose statin therapy. Initial light microscopy with sparse endomysial lymphocytic infiltrates could be consistent with an immune mediated necrotizing myopathy; however inflammatory infiltrates have also been noted in cases of SLONM [1]. Additionally, there were no additional findings to suggest the presence of an alternative inflammatory myopathy (i.e. perifascicular muscle fiber atrophy, rimmed vacuoles, and/or ragged red fibers). When taken into context, the presence of nemaline fibers on electron microscopy was most supportive of the diagnosis of SLONM. Cases of concurrent immune mediated necrotizing myopathies related to HMGCR antibodies and SLONM have not been described in the existing literature, although it is possible that both conditions were present in our patient.

**Fig. 1:**
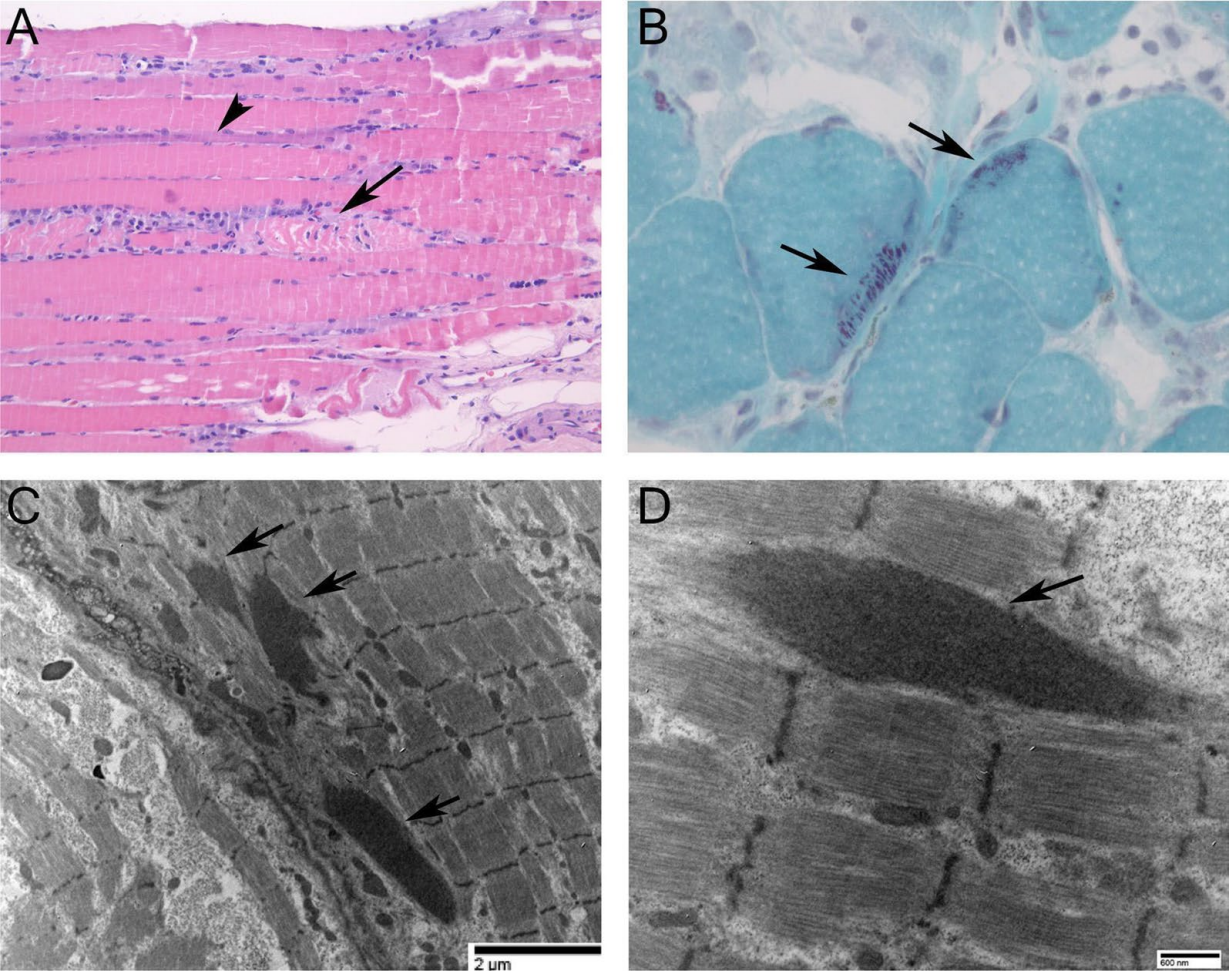
Muscle Biopsy. **A** Hematoxylin and eosin stain showing necrotic (arrow) and regenerating (arrowhead) muscle fibers. (Original magnification 200X) **B** Nemaline bodies (arrows) on modified Gomori trichrome stain (400X). **C**, **D**. Nemaline bodies (arrows) on transmission electron micrographs. (67,000X and 20,000X, respectively)

While diagnosis of SLONM can be readily confirmed by biopsy of an affected muscle, it is important to alert the pathologist of the clinical suspicion of this diagnosis as nemaline bodies can be easily overlooked due to their small size of less than 1 µm in length. Necrotic and regenerating muscle fibers are demonstrated via light microscopy in Fig. 1A. Nemaline bodies can be seen as short red linear inclusions on a modified Gomori trichrome stain on cryostat sections shown in Fig. 1B. Confirmation requires electron microscopy as seen in Fig. 1C, D. In the absence of electron microscopy, the identity of nemaline bodies can be supported by immunostaining for myotilin34 and actinin35 [2]. Lastly, all patients with a suspected diagnosis of nemaline myopathy should be tested for HIV, since a similar myopathy can manifest early in the infection before other manifestations of advanced HIV are evident.

Subacute to chronic progressive weakness, elevated CK and aldolase levels and subsequent ventilator dependent respiratory failure were all suggestive of a neuromuscular disease (NMD) in our patient. Respiratory failure continues to be a leading cause of morbidity and mortality in neurologic disease [3]. Neuromuscular pathology can be anatomically characterized by diseases affecting either the central nervous system (upper or lower motor neuron diseases), peripheral nervous system, neuromuscular junction or skeletal muscle [4]. Respiratory muscle weakness can impair ventilation causing hypercarbia and resultant hypoxemia. Related symptomatology includes daytime somnolence, insomnia, dyspnea, orthopnea and diminished cough. Bulbar muscle involvement can produce dysphagia, dysarthria, facial asymmetry and inability to manage secretions. As a result, patients with chronic NMDs are vulnerable to a host of respiratory complications including recurrent aspiration, atelectasis and lower respiratory tract infections.

Our patient’s unique course prior to hospitalization, proximal muscle weakness without sensory deficit, and elevated muscle related enzymes all suggested skeletal muscle disease as a potential culprit. Myopathy may be suspected with isolated muscular weakness without upper or lower motor neuron signs and can be further delineated based on the presence of extramuscular symptoms and risk factors. Common risk factors include medications and illicit drug exposure, genetics, infection, metabolic disturbances, rash or a host of connective tissue diseases. Common causes of myopathy associated with respiratory failure are listed below in Table 1 [5–12].

**Table 1:** Causes of myopathy associated with respiratory failure

Causes of myopathy associated with respiratory failure	
**Inflammatory**	**Electrolyte disorders**
Polymyositis, dermatomyositis	Hypokalemia
Inclusion body myositis	Hypophosphatemia
Systemic lupus erythematosus, rheumatoid arthritis, Sjogren’s Syndrome, Overlap Syndromes	**Viral myositis**
**Endocrine**	**Drug/toxin**
Hypothyroidism	Cocaine, heroin, alcohol
Cushing’s syndrome	Corticosteroids
**Inherited myopathies**	HMG-CoA reductase inhibitors, SSRIs, zidovudine
Acid maltase deficiency	**Other**
Muscular dystrophy	Sarcoidosis
**Critical illness**	Amyloidosis
Critical illness, sepsis, ventilator-associated injury	Paraneoplastic syndromes

While critical illness was undoubtedly a contributing factor to our patient’s functional decline, this would not explain his preceding symptoms or findings on muscle biopsy. In addition to histopathological examination, electrophysiological studies (EMG) and imaging (MRI) can also be utilized and may provide benefit in characterizing undifferentiated disease (i.e. lower motor neuron, neuromuscular junction, muscle) or identifying optimal sites for biopsy [13]. Though technical challenges with these modalities may arise in the ICU setting due to artifacts from neighboring devices, inability to maintain technical standards, and inability to voluntarily cooperate with testing [13]. Genetic testing may also be appropriate when considering heritable muscular dystrophies and myopathies.

Pulmonary function testing is integral to the workup of neuromuscular disease. Inspiratory muscle weakness and failure to expand the thoracic cage leads to increased chest wall elastance and reduced compliance. The resultant restrictive pattern on pulmonary function testing will result in reduced vital capacity (VC) and total lung capacity (TLC) with preserved diffusion capacity of carbon monoxide (DLCO). Expiratory muscle involvement further reduces VC and may result in reduced expiratory residual volume (ERV) with increased residual volume (RV) and RV/TLC ratio. Patients with significant bilateral diaphragmatic weakness become reliant on gravity to support inspiration and may develop postural reduction of VC in excess of 30–50% when moving from the upright to supine position [14]. Further testing of respiratory muscle strength includes maximal inspiratory pressure (MIP or PImax), sniff nasal inspiratory pressure (SNIP) and maximal expiratory pressure (MEP or PEmax). While these tests can be considered to support a diagnosis, these tests are more commonly used to monitor disease activity and predict one’s risk of developing respiratory failure. Fluoroscopic “sniff” testing can be misleading in conditions causing bilateral diaphragmatic weakness due to high rates of false negative testing [15].

Long-term invasive or noninvasive ventilation (NIV) is necessary to support respiratory muscle function and progressive respiratory failure. Numerous indicators to initiate ventilatory support in chronic neuromuscular disease have been proposed, including demonstration of nocturnal hypercapnia. Nutritional support, respiratory physiotherapy and effective cough assistance also show varied benefit in patients with chronic neuromuscular disease [3]. Currently, no consensus recommendations exist to guide additional management of SLONM. The use of immunosuppressive agents, intravenous immunoglobulins and plasmapheresis have been reported [1]. Additionally, peripheral blood stem cell transplantation has led to improvement in a small series of patients with SLONM associated with MGUS [16, 17]. Further supportive interventions include respiratory physiotherapy and the use of non-invasive ventilation.

## Data Availability

Not applicable.

## Abbreviations

SLONM: Sporadic late onset nemaline myopathy
ICU: Intensive care unit
CK: Creatinine kinase
U/L: Units per liter
CT: Computed tomography
MGUS: Monoclonal gammopathy of undetermined significance
EMG: Electromyography
MRI: Magnetic resonance imaging
NMD: Neuromuscular disease
HMGCR: 3-Hydroxy-3-methyl-glutaryl-coenzyme A reductase
MIP: Maximal inspiratory pressure
SNIP: Sniff nasal inspiratory pressure
MEP: Maximal expiratory pressure
NIV: Non-invasive ventilation
VC: Vital capacity
TLC: Total lung capacity
DLCO: Diffusion capacity of carbon monoxide
RV: Residual volume
ERV: Expiratory residual volume
